# Remnant cholesterol, not LDL cholesterol, is associated with the risk of hypertension in the middle-aged and elderly population: Bushehr Elderly Health cohort

**DOI:** 10.3389/fcvm.2025.1483778

**Published:** 2025-10-03

**Authors:** Behnaz Esmaeili, Shahnaz Esmaeili, Noushin Fahimfar, Mostafa Rezaei Tavirani, Farideh Razi, Iraj Nabipour, Patricia Khashayar, Fatemeh Bandarian

**Affiliations:** ^1^ Department of Basic Medical Sciences, Khoy University of Medical Sciences, Khoy, Iran; ^2^ Department of Basic Sciences/Medical Surgical Nursing, School of Nursing and Midwifery, Tehran University of Medical Sciences, Tehran, Iran; ^3^ Osteoporosis Research Center, Endocrinology and Metabolism Clinical Sciences Institute, Tehran University of Medical Sciences, Tehran, Iran; ^4^ Proteomics Research Center, Shahid Beheshti University of Medical Sciences, Tehran, Iran; ^5^ Endocrinology and Metabolism Research Center, Endocrinology and Metabolism Clinical Sciences Institute, Tehran University of Medical Sciences, Tehran, Iran; ^6^ The Persian Gulf Marine Biotechnology Research Center, The Persian Gulf Biomedical Sciences Research Institute, Bushehr University of Medical Sciences, Bushehr, Iran; ^7^ Department of Chemistry, Ghent University, Ghent, Belgium; ^8^ International Institute for Biosensing, University of Minnesota, Minneapolis, MN, United States; ^9^ Metabolomics and Genomics Research Center, Endocrinology and Metabolism Molecular-Cellular Sciences Institute, Tehran University of Medical Sciences, Tehran, Iran

**Keywords:** hypertension, remnant cholesterol, elderly peoples, lipid profile, dyslipidemia

## Abstract

**Background:**

Despite available studies, the link between dyslipidemia and hypertension remains unclear, particularly among different ethnicities, age groups, and genders. This study aimed to assess this association in the Iranian elderly population.

**Methods:**

In a cross-sectional study, we used data from phase II of the Bushehr Elderly Health Program (BEHP). Participants were divided into hypertensive and non-hypertensive groups based on their blood pressure. The association between lipid parameters and hypertension (HTN) was evaluated using multiple logistic regression analysis. Additionally, we conducted a subgroup analysis by gender, age, and BMI (Body Mass Index).

**Results:**

In this study, 1,918 people with a mean age of 62.10 ± 8.05 were included. Of them, 1,133 (59.1%) were hypertensive, and 57.2% were females (1,097). Among the lipid profiles, individuals with HTN had higher levels of triglyceride (TG) and remnant cholesterol (RC). There was a negative association between high-density lipoprotein (HDL-c) levels and high blood pressure 0.987(0.978–0.995), p:0.003, and a positive association between TG and RC ≥ 30 levels and HTN ([1.003(1.002–1.005), *p* < 0.001]; [1.36 (1.123–1.648), p:0.002], respectively). This positive association was observed after full adjustment for age, gender, and BMI. While HDL-C was lower and RC levels were significantly higher only in the hypertensive people aged ≤65 (*p* < 0.01), higher RC levels were observed in the hypertensive groups independent of BMI levels (*p* = 0.004). Moreover, the levels of RC ≥ 30 mg/dl were associated with the risk of HTN only in males.

**Conclusion:**

In the middle-aged and elderly hypertension population, there is a positive and statistically significant association between RC and HTN. This suggests that this indicator is associated with HTN, particularly in those with normal or subnormal levels of traditional risk factors. Moreover, this association may be affected by age and anthropometric parameters as well as life style factors.

## Introduction

High blood pressure (hypertension/HTN) is a major global health challenge with a growing incidence and prevalence, affecting approximately 30% of adults worldwide (1–4). It is a main risk factor for cardiovascular diseases and the third leading cause of disability (3, 5). In Iran, its prevalence among adults aged 25–70 years is approximately 25.6% (6). Therefore, identifying the associated risk factors is important for reducing the burden of this disease.

Several risk factors, such as low physical activity, smoking, and metabolic disorders like dyslipidemia, can increase the risk of HTN (7, 8). Among these factors, both HTN and dyslipidemia share the same pathophysiological mechanisms and have a synergistic effect, increasing the risk of cardiovascular events (9). In this regard, numerous investigations have been conducted on the relationship between HTN and blood lipid profile, as well as its underlying mechanisms (10–14).

While most of the investigations have evaluated the role of traditional lipid parameters (e.g., total cholesterol (Tch), low-density lipoprotein (LDL), high-density lipoprotein (HDL), and triglyceride (TG)) in HTN, recent studies focused on the effects of non-traditional lipid parameters such as remnant cholesterol (RC) in this context (15, 16). Remnant cholesterol (RC) is the cholesterol content of lipoproteins with a high concentration of triglycerides and is made of very-low-density lipoprotein (VLDL), intermediate-density lipoprotein (IDL), and chylomicron remnants (17, 18).

Increasing evidence shows the association of RC with cardiovascular risk and HTN development (15, 19). More interestingly, the relationship between RC and HTN is independent of LDL-C levels (20), and its management has been proposed as a new way of controlling or treating hypertensive patients (21). Although the exact mechanisms of this association are not known, evidence shows that RC has more deteriorative effects on vascular damage than traditional lipids (22, 23). In this regard, the established role of RC in inflammation, the endothelial and endocrine system (15, 23–27), has been illustrated by several investigations.

Despite the available studies, the evidence regarding the association of dyslipidemia with HTN is still uncertain, especially across different ethnicities, age groups, and sexes. Therefore, we aimed to assess the relationship between lipid parameters, particularly RC, and HTN in the middle-aged and elderly Iranian population. To the best of our knowledge, no study has investigated the association of RC with HTN in Iranian middle-aged and elderly populations.

## Material methods

In this cross-sectional study, we used data from phase II of the Bushehr Elderly Health Program (BEHP), a community-based prospective cohort study with 3,000 participants. This program aimed to assess the prevalence of non-communicable disease in the middle-aged population. The PoCOsteo study is an extension to the second stage of BEH, including 2,000 people aged more than 50 years, focusing on musculoskeletal diseases (28). A multistage stratified random sampling method was used to recruit participants. This study was approved by the Research Ethics Committee of the Endocrinology & Metabolism Research Institute (Ethical code: IR.TUMS.EMRI.REC.1394.0036). Also, informed consent was obtained from all participants before enrollment.

Information related to the study's design, clinical examination, data collection, biochemical measurements, and methodology can be found elsewhere (28–30). People with systolic blood pressure >140, or diastolic blood pressure >90 (according to the WHO definition) (31), or those taking blood pressure medication were considered hypertensive individuals. We excluded individuals with incomplete data from the study. After measurement of blood lipid and lipoproteins, we used the following formula for the calculation of RC values: total cholesterol minus LDL-cholesterol minus HDL-cholesterol (32, 33).

### Data analysis

Demographic characteristics including age, gender, body max index (BMI), hip circumference (HC), waist circumference (WC), diastolic blood pressure (DBP), and systolic blood pressure (SBP) were collected and reported as mean ± standard deviation (SD) (for quantitative variables) and frequency with the number and percentage (for categorical variables). The lipid profile, including total cholesterol (TC), high-density lipoprotein (HDL-C), low-density lipoprotein (LDL-C), and remnant cholesterol (RC), as well as other laboratory parameters, including fasting blood sugar (FBS), hemoglobin A1c (HbA1c), was also measured. To reduce RC misestimate, LDL was measured by direct method.

Differences in continuous variables between the two groups were examined by an independent t-test or Mann–Whitney test as appropriate. The chi-square test was used to examine the significant relationship between two qualitative variables. Moreover, we performed a subgroup analysis by age (Age ≤65 and Age >65) and BMI (BMI < 30 and BMI ≥30 kg/m^2^). Then, multivariate logistic regression was used to estimate the relationship between various parameters and HTN and to remove confounders. In the crude model, no variables were adjusted, and in model 1, data was adjusted for age, gender, BMI, smoking and lipid -lowering drug use. For statistical analysis, we used the SPSS software version 23.0 for Windows (IBM, Armonk, New York). A value of *p* < 0.05 was considered statistically significant.

## Results

### Demographic characteristics of study participants

Generally, 1,918 people with a mean age of 62.10 ± 8.05 years were included in the study, of whom 1,133 (59.1%) were hypertensive, and 57.2% were females (1,097). The baseline characteristics of the participants are presented in Table 1. Individuals in the HTN group had higher values for age, BMI, FBS, WC, HC, and HbA1c (%), and a lower percentage of current smokers. The non-hypertension group exhibited higher levels of TC, HDL-c, LDL-C, and non-HDL-c (*p* < 0.001), whereas hypertensive individuals showed higher levels of RC and TG (*p* < 0.01). Moreover, the usage of lipid-lowering agents was higher in hypertensive individuals compared to non-hypertensive individuals (*p* < 0.001) (Table 1).

**Table 1:** Demographic characteristics and laboratory findings of participants in the hypertensive group compared with the non-hypertensive group.

Characteristics			Total	Hypertension	Non-hypertension	*P*-value
Number (%)			1,918 (100)	1,133 (59.1)	785 (40.9)	–
Demographic variables	Sex	Men (%)	821 (42.8)	499 (44)	322 (41)	0.18
		Women (%)	1,097 (57.2)	643 (56)	463 (59)	
	Age (years) (mean ± SD)		62.10 ± 8.05	63.89 ± 8.23	59.94 ± 7.28	<0.001*
Lifestyle factors	Current smoking, *n* (%)		440 (22.9)	219 (19.4)	221 (28.2)	<0.001*
Medication use	Lipid-lowering agents use, *n* (%)		552 (28.8)	412 (36.4)	140 (17.8)	<0.001*
Anthropometric	BMI (kg/m^2^) (mean ± SD)		28.21 ± 4.92	28.70 ± 4.93	27.51 ± 4.81	<0.001*
	WC (Cm) (mean ± SD)		99.97 ± 11.22	101.68 ± 10.94	97.48 ± 11.15	<0.001*
	HC (Cm) (mean ± SD)		105.71 ± 9.92	106.49 ± 10.02	104.59 ± 9.67	<0.001*
Cardiovascular risk factors	SBP (mmHg) (mean ± SD)		133.10 ± 20.49	142.85 ± 19.34	119.03 ± 12.28	<0.001*
	DBP (mmHg) (mean ± SD)		80.81 ± 10.63	84.23 ± 11.22	75.86 ± 7.30	<0.001*
Laboratory tests	FBS (mg/dl) (mean ± SD)		110.60 ± 42.87	114.10 ± 42.90	105.56 ± 42.35	<0.001*
	HbA1c (%)(mean ± SD)		6.31 ± 1.48	6.46 ± 1.52	6.07 ± 1.39	<0.001*
	TC (mg/dl) (mean ± SD)		187.11 ± 41.64	183.56 ± 42.59	192.23 ± 39.71	<0.001*
	TG (mg/dl) (mean ± SD)		148.81 ± 81.36	156.32 ± 89.51	137.96 ± 66.45	<0.001*
	LDL-C (mg/dl) (mean ± SD)		101.20 ± 3,398	96.90 ± 34.34	107.40 ± 32.50	<0.001*
	HDL-C (mg/dl) (mean ± SD)		49.14 ± 11.39	48.40 ± 11.29	50.20 ± 11.46	<0.001*
	Non_HDL (mg/dl) (mean ± SD)		137.97 ± 40.73	135.15 ± 41.87	142.03 ± 38.68	<0.001
	RC (mg/dl) (mean ± SD)		36.77 ± 28.38	38.25 ± 28.85	34.63 ± 27.58	0.006*

* *p* < 0.05 significant.

FBS, fasting blood sugar; RC, remnant cholesterol; SBP, systolic blood pressure; DBP, diastolic blood pressure; WC, waist circumference; HC, hip circumference; TG, triglyceride; Tch, total cholesterol.

### Differences in lipid profiles between hypertensive and non-hypertensive groups based on age and BMI

We performed a subgroup analysis according to age and BMI as shown in Table 2. In both the Age ≤65 and Age >65 groups, we found that TC and LDL-C were higher, and TG levels were lower in the non-hypertensive individuals. Moreover, HDL-c was lower and RC levels were significantly higher only in the hypertensive people aged ≤65 (*p* < 0.01). In both the BMI ≤ 30 and BMI >30 kg/m^2^ groups, TC and LDL-C were higher, and TG levels were lower in non-hypertensive people (*p* < 0.01). Remnant cholesterol was significantly higher in the hypertensive group in individuals with BMI < 30 kg/m^2^ and BMI ≥30 kg/m^2^ groups (*p* = 0.04).

**Table 2:** Subgroup analysis of lipid profile differences between hypertension and non-hypertension according to age and BMI.

Parameter	Non-hypertension *N* = 607	Hypertension *N* = 678	*p*-value	Non-hypertension *N* = 178	Hypertension *N* = 455	*p*-value
	Age ≤ 65 *N* = 1,285			Age > 65 *N* = 633		
TC (mg/dl)(mean ± SD)	193.13 ± 40.24	186.57 ± 41.65	0.004*	189.18 ± 37.78	179.06 ± 43.62	0.004*
TG (mg/dl) (mean ± SD)	141.62 ± 68.41	166.12 ± 92.31	<0.001*	125.47 ± 57.75	141.73 ± 83.16	0.005*
LDL (mg/dl) (mean ± SD)	108.24 ± 32.81	98.34 ± 34.39	<0.001*	104.54 ± 31.30	94.62 ± 34.17	<0.001*
HDL (mg/dl) (mean ± SD)	50.07 ± 11.40	47.85 ± 11.34	<0.001*	50.62 ± 11.71	49.22 ± 11.17	0.16
Non-HDL (mg/dl) (mean ± SD)	143.05 ± 39.08	138.72 ± 40.90	0.052	138.56 ± 37.20	129.84 ± 42.77	0.012*
RC (mg/dl) (mean ± SD)	34.81 ± 27.40	40.28 ± 29.82	<0.001*	34.02 ± 28.27	35.22 ± 27.08	0.62
Parameter	Non-hypertension *N* = 559	Hypertension *N* = 749	*p*-value	Non-hypertension *N* = 226	Hypertension *N* = 384	*p*-value
	BMI < 30 *N* = 1,308			BMI ≥ 30 *N* = 610		
TC (mg/dl) (mean ± SD)	191.66 ± 40.09	182.48 ± 41.25	<0.001*	193.64 ± 38.80	185.65 ± 45.07	0.021*
TG (mg/dl) (mean ± SD)	134.99 ± 66.15	154.21 ± 93.37	<0.001*	144.29 ± 66.78	160.43 ± 81.43	0.013*
LDL (mg/dl) (mean ± SD)	106.70 ± 31.51	95.89 ± 33.93	<0.001*	109.13 ± 34.84	98.87 ± 35.09	<0.001*
HDL (mg/dl) (mean ± SD)	49.79 ± 11.35	48.16 ± 11.60	0.011*	51.20 ± 11.71	48.88 ± 10.65	0.013*
Non-HDL (mg/dl) (mean ± SD)	141.87 ± 38.85	134.32 ± 40.62	<0.001*	142.43 ± 38.33	136.77 ± 44.23	0.097
RC (mg/dl) (mean ± SD)	35.17 ± 27.81	38.43 ± 29.67	0.044*	33.31 ± 27.02	37.90 ± 27.21	0.044*

*Means significant difference.

### Multivariate logistic regression analysis of lipid profile and prevalence of HTN

Based on the crude model in the entire population, an increase in RC ≥ 30 and TG levels was associated with increased risk of HTN [1.390 (1.145–1.686)], *p* = 0.001) and [3.042 (1.865–4.961), *p* < 0.001], respectively. This positive association was observed after adjustment for age, gender, BMI, smoking and lipid-lowering drug use. Both unadjusted and adjusted models showed that an increase in HDL-c exhibited a negative trend with the risk of HTN and reduced the risk of HTN, as shown in Table 3. Relation of other lipid factors including TC, LDL-C and non-HDL-C with HTN are shown in Table 3, also.

**Table 3:** Association between lipid profile and HTN in the whole population.

Lipid parameter	Unadjusted OR (95% CI) *P* value	Adjusted Model 1^a^ OR (95% CI) *P* value
HDL-C increase	0.986 (0.975–0.996), *p*:0.007	0.983 (0.974–0.991), *p* < 0.001
TC Increase	0.995 (0.993–.0,997), *p* < 0.001	-removed from equation
LDL Increase	0.991 (0.988–0.994), *p* < 0.001	0.995 (0.992–0.998), *p* = 0.001
Triglyceride increase	2.907 (1.847–4.576), *p* < 0.001	3.042 (1.865–4.961), *p* < 0.001
non-HDL-C increase	0.996 (0.994–0.998), *p* < 0.001	-removed from equation
RC increase ≥30 mg/dl	1.310 (1.091–1.573), *p*: 0.004	1.390 (1.145–1.686), *p* = 0.001

^a^ Adjusted for age, gender, and BMI, smoking and lipid lowering drug use.

In males, before and after adjusting for age, BMI, smoking and lipid-lowering drug use, an increase in RC ≥ 30 increased the risk of HTN [1.430 (1.079–1.898), *p*: 0.013] and [1.572 (1.169–2.113), *p* = 0.003], respectively (Table 4). In females, before and after adjusting for age, BMI, smoking and lipid-lowering drug use there was no significant association between increase in RC ≥ 30 and the risk of HTN (Table 5).

**Table 4:** Association between lipid profile and HTN in males.

Lipid parameter	Unadjusted OR (95% CI) *P* value	Adjusted Model 1^a^ OR (95% CI) *P* value
HDL-C increase	0.989 (0.975–1.003), p:0.11	-removed from equation
TC Increase	0.997 (0.993–1.001), p: 0.09	-removed from equation
LDL Increase	0.992 (0.988–0.996), *p* < 0.001	-removed from equation
Triglyceride increase	2.942 (1.492–5.801), *p* = 0.002	3.505 (1.654–7.428), *p* = 0.001
non-HDL-C increase	0.998 (0.994–1.001), p:0.19	-removed from equation
RC increase ≥30 mg/dl	1.430 (1.079–1.898), p: 0.013	1.572 (1.169–2.113), *p*: 0.003

^a^ Adjusted for age, BMI, smoking and lipid lowering drug use.

**Table 5:** Association between lipid profile and HTN in females.

Lipid parameter	Unadjusted OR (95% CI) *P* value	Adjusted Model 1^a^ OR (95% CI) *P* value
HDL-C increase	0.982 (0.975–0.996), *p* = 0.007	0.984 (0.9,734–0.9956), *p* = 0.005
TC increase	0.994 (0.991–0.997), *p* < 0.001	0.997 (0.994–1.000), *p* < 0.046
LDL increase	0.990 (0.987–0.994), *p* < 0.001	0.994 (0.990–0.998), *p* = 0.002
Triglyceride increase	2.870 (1.559–5.286), *p* = 0.001	2.441 (1.268–4.698), *p* = 0.008
non-HDL-C increase	0.995 (0.992–0.998), *p* < 0.001	-removed from equation
RC increase ≥30 mg/dl	1.23 (0.968–1.569), *p* = 0.09	-removed from equation

^a^ Adjusted for age and BMI, smoking and lipid lowering drug use.

## Discussion

In this study, we evaluated the association between lipid parameters, particularly RC, and HTN. We found that in the overall population, hypertensive people were older individuals with higher levels of anthropometric parameters, TGs, and RC. In the subgroup analysis based on the age, only hypertensive people with aged ≤65 had significantly higher levels of RC. Our logistic regression analyses revealed that the risk of HTN increased significantly with an increase in RC ≥ 30 and TG levels.

Even though the available evidence has highlighted the relationship between lipid profile and HTN, the pattern of lipid parameters associated with HTN varies considerably among the studies. While Deng et al. showed a positive association between TC, LDL-C, and non-HDL-C levels and HTN, there was no significant association between the levels of TGs and HTN among Chinese women (11). Similar results were obtained in a study conducted on Chinese adult males (9). In the Iranian population, a high level of TG in females was associated with HTN (34). In another study, Tohidi et al, showed TG levels were useful indicators of HTN rather than Tch and non-HDL-c levels (35). While the exact reasons for these inconsistent results remain unknown, they may be attributed to age, genetic differences, and lifestyle of the participants (11).

In line with a study conducted on Chinese women (11), the results of this study showed a negative association between HTN and HDL-c levels. HDL-c has anti-inflammatory and vasodilator effects. Its abnormal levels are linked to endothelial dysfunction (36, 37), supporting its association with blood pressure. However, some reports indicated a positive relationship between this indicator and HTN (9, 38). Although most investigations indicated higher levels of Tch and LDL-C in hypertensive people (9, 11), our results showed lower levels of these indicators among hypertensive individuals. These differences may be associated with the age of the participants in this study. Aging has a decreasing effect on LDL-C and TC levels (39). On the other hand, most of the hypertensive participants were lipid-lowering drug users. To verify the findings of this study, another study is required to account for confounding variables, including the impact of treatment on lipid profile levels.

Risk factors such as dyslipidemia and BMI status are the same between HTN and metabolic syndrome, suggesting the pathophysiological mechanisms involved in the development of HTN may be similar to those involved in the metabolic syndrome (9). In both of them, endothelial dysfunction plays an important role (40, 41). Moreover, there is a relationship between BMI and TG levels (42). Even at normal levels of cholesterol, hypertriglyceridemia can increase the risk of cardiovascular diseases (43).

In this study, despite a reduction in Tch and LDL-C levels, the levels of TGs and anthropometric parameters were higher in people with HTN, which shows that in the Iranian elderly, the characteristic indicators of metabolic syndrome may play a more important role in HTN development, which needs further evaluation.

Despite the controversial results related to the relationship of conventional lipid parameters with HTN, most recent studies have shown a positive and significant association between RC and high blood pressure in different populations (15, 20, 24, 27). Recent investigations have suggested that managing RC levels could efficiently reduce persistent residual cardiovascular risk (44, 45). Therefore, numerous studies evaluated and compared the role of this indicator to other conventional lipid parameters in HTN development. The results of these studies revealed that compared with TGs, LDL-C, and Tch levels, the levels of RC had the strongest association with HTN (15, 24). Moreover, this association was stronger in the high RC/low LDL-C group, suggesting the role of RC in HTN incidence beyond traditional cardiovascular risk factors (46).

In our study, the levels of RC were significantly higher in the HTN group. RC serves as a valuable biomarker for assessing hypertension risk in the elderly, which supports longitudinal studies.

Moreover, in the entire population, a positive association was found between RC and HTN, even after adjusting for confounders. In subgroup analysis based on gender, while this association was significant only in males, there was a positive relationship between these parameters after adjusting for age and BMI in females (Tables 4, 5). Consistent with these results, Li et al. showed that the levels of RC were higher in Chinese males with higher BMI. They indicated that among lipid parameters, RC had the highest association with central systolic blood pressure independently of other lipid parameters. This association may be due to the physiological characteristics of this cholesterol (24) and the fact that the cholesterol in residual lipoproteins has an atherogenic and proinflammatory property more than that of cholesterol in LDL-C (19, 47). Kasahara et al. also showed the levels of remnant-like lipoprotein particle cholesterol (RLP-C) were higher in males (48). However, some reports showed females had higher RC levels (20, 27) and this association may be related to the impacts of sex hormones on cholesterol metabolism (49).

Most of the evidence indicated the impact of age, gender, and BMI on the incidence and prevalence of HTN among the participants. Among these factors, aging is a well-documented risk factor (34) due to its role in vascular stiffness (50). However, there are inconsistent results about the impact of gender on the HTN risk (34, 51). While aging increases the incidence rate in both sexes, the prevalence of HTN is higher in males than in females among younger adults (less than 40 years old) (52). In our study, we found that differences in the RC levels were significant only in the hypertensive individuals aged ≤65. In line with these results, Alijanvand et al. demonstrated that the association between RC and HTN increased with obesity in the high-risk group (53). Moreover, Shi et al. showed that the participants in the low LDL-c/high RC group were younger and females with higher BMI and TG levels. They declared that increased levels of RC were linked to HTN independent of LDL levels (20). Wu et al. also demonstrated that RC levels through inflammatory pathways increase the risk of HTN even in normal levels of TC, LDLC, and HDL-C (27). These results show that remnant cholesterol has an impressive effect on the development of HTN however this effect may be affected by demographic and anthropometric characteristics of the individuals. Moreover, in the assessment of the HTN risk, we should pay more attention to the levels of RC of the participants, particularly in those with normal or subnormal LDL-C levels.

## Limitations

We acknowledge several limitations of the present study. Firstly, we were unable to draw causal conclusions due to the cross-sectional nature of the study. Secondly, we obtained the RC values through indirect measurements with calculation by formula which may introduce bias due to inherent assumptions about the relationship between triglycerides and remnant lipoproteins which can lead to overestimating or underestimating the relationship.

## Conclusion

In conclusion, although the levels of traditional lipid parameters were below the normal, the levels of RC and TG were higher in the group with HTN, and an increase in these indicators was associated with the risk of developing HTN. This shows the superior ability of remnant cholesterol in assessing the risk of HTN compared to traditional lipid parameters. Furthermore, this association may be more significant in younger adults with higher anthropometric characteristics.

## Data Availability

The raw data supporting the conclusions of this article will be made available by the authors, without undue reservation.
